# Environmentally Sustainable and Ecosafe Polysaccharide-Based Materials for Water *Nano*-Treatment: An Eco-Design Study

**DOI:** 10.3390/ma11071228

**Published:** 2018-07-17

**Authors:** Ilaria Corsi, Andrea Fiorati, Giacomo Grassi, Irene Bartolozzi, Tiberio Daddi, Lucio Melone, Carlo Punta

**Affiliations:** 1 Department of Physical, Earth and Environmental Sciences, University of Siena, Via Mattioli 4, 53100 Siena, Italy; giacomograssi6@gmail.com; 2 Department of Chemistry, Materials, and Chemical Engineering “G. Natta” Politecnico di Milano, Via Mancinelli 7, 20131 Milano, Italy; andrea.fiorati@polimi.it (A.F.); lucio.melone@polimi.it (L.M.); 3 Sant’Anna School of Advanced Studies, Institute of Management, Piazza Martiri della Libertà 33, 56127 Pisa, Italy; irene.bartolozzi@sssup.it (I.B.); tiberio.daddi@sssup.it (T.D.); 4 Ergo S.r.l., c/o Technology Centre, Via Giuntini 25/29–int. 29, 56023 Pisa, Italy

**Keywords:** polysaccharides, nanocellulose, nanostructured materials, ecosafety, LCA

## Abstract

Nanoremediation, which is the use of nanoparticles and nanomaterials for environmental remediation, is widely explored and proposed for preservation of ecosystems that suffer from the increase in human population, pollution, and urbanization. We herein report a critical analysis of nanotechnologies for water remediation by assessing their sustainability in terms of efficient removal of pollutants, appropriate methods for monitoring their effectiveness, and protocols for the evaluation of any potential environmental risks. Our purpose is to furnish fruitful guidelines for sustainable water management, able to promote nanoremediation also at European level. In this context, we describe new nanostructured polysaccharide-based materials obtained from renewable resources as alternative efficient and ecosafe solutions for water nano-treatment. We also provide eco-design indications to improve the sustainability of the production of these materials, based on life-cycle assessment methodology.

## 1. Introduction

The increasing and rapid deterioration and degradation of the water quality is one of the most challenging issues facing the 21st century. It can be ascribed to a variety of factors such as population growth, the effects of climate change on the hydrologic cycle and increasing pollution. Globally, extensive research has been performed to address such urgent environmental issues and new technologies have been developed to remediate water pollution by both organic and inorganic contaminants. However, the cost and challenge associated with the treatment of both groundwater and wastewater and the increasing awareness of environmental risks calls for continued improvement and innovation. Nanotechnology has significantly contributed to remarkable industrial and societal changes over recent decades. Among the wide variety of fields of application of nanotechnology, considerable efforts have been devoted to exploiting the potential of engineered nanomaterials (ENMs) for environmental remediation, commonly referred to as “nanoremediation” [1]. Compared to conventional in situ remediation techniques, such as thermal treatment, air sparging, chemical oxidation and bioremediation, often coupled with on-site pump-and-treat processes [2], which are known to be expensive, partially effective and time-consuming, nanoremediation has emerged as a new clean-up method that is less costly, more effective as well as environmentally, socially, and economically sustainable [3,4]. Indeed, nanotechnologies allow treatment of contaminated media in situ and minimize the addition of further chemicals in the clean-up process [5]. The unique properties of ENMs are particularly advantageous for large-scale in situ remediation of contaminated waters and have certainly boosted the efficiency of nanotechnology-based decontamination strategies, compared to “conventional” approaches [6,7]. ENMs, due to their nanometric size, present a very high and reactive surface area, compared to the same volume of bulk material. They can also be tuned with desired properties by tailoring the synthetic processes to meet case-specific needs and overcome applicative limitations stemming from the complexity of environmental matrices to be treated. Additionally, the far-reaching mobility of ENMs in aquatic media maximizes their potential for treating large volumes of contaminated environmental matrices [8]. According to the Project of Environmental Nanotechnology web site and United States Environmental Protection Agency (USEPA), in the last ten years, almost 70 sites have been successfully treated worldwide at field scale, by using nanoremediation techniques. These approaches have significantly reduced time frame (days vs. months) and operational costs (up to 80%) in comparison with conventional methods [4,9]. It has been estimated that there are more than 2.5 million potentially polluted sites in Europe which need to be remediated, and that 350,000 contaminated sites could represent a potential risk to humans or the environment [10]. Moreover, a remediation technology must attend to cost-benefit approaches considering practical immediate issues and long-term expectancies.

Despite such promising expectations, environmental and human risk assessment associated with the use of ENMs is still a matter of debate and nanoremediation is seen still as an emerging technology [6]. It has been slowly applied in Europe [11] probably because of the emerging societal worries on nanotechnology and the current lack of regulatory and proper legislative supports [12,13,14].

We herein report a critical analysis on the use of the ENMs for water remediation, with the aim of sharing the strategy developed within the NanoBonD project (Nanomaterials for Remediation of Environmental Matrices associated to Dewatering), funded in the framework POR CReO FESR Tuscany 2014–2020, whose objective is the development of innovative, ecofriendly and ecosafe polysaccharide-based nanotechnologies for the remediation of contaminated sediments and waters.

## 2. Eco-Design of ENMs for Environmental Remediation

The particularly desirable ENMs characteristics, which make them suitable for environmental remediation, can negatively rebound of the safety of application of such materials in water remediation [15]. The current debate relies on the balance between known benefits of nanoremediation and potential risks associated to the use of ENMs in natural environments mainly due to their mobility, transformations and ultimately potential ecotoxicity [16]. Costs and benefits are not always easy to handle especially for emerging materials, at least at the beginning when unexplored aspects are still present and contradictory results exist considering both human health and environmental effects. Certainly, some concerns occur regarding their use in contaminated water bodies and potential “side effects”: once dispersed their mobility could increase the ability to be up taken by plants or animals at the site or further away and adversely affect them. The mobility, small size, and overall reactivity of ENMs can therefore dramatically improve the transport of ENMs in water compartments, as they could potentially reach undesired targets and lead to hazardous effects. Additionally, a typical nanoremediation process entails that ENMs are dispersed in environmental compartments, such as ground or surface waters, which are defined by peculiar levels of ionic strength, dissolved oxygen and dissolved organic matter (DOM) contents, and other physico-chemical parameters. The interactions of ENMs with such complex media can alter significantly both the chemical and physical nature of the “as-manufactured” materials and led to the formation of significantly different “weathered” or “aged” species [17]. As an example, silver [18,19,20] and copper [21] nanoparticles are converted into the corresponding sulfides species via sulfidation processes, once dispersed in natural waters. The dispersion quality of ENMs can be equally affected, often leading either to homo- and hetero-aggregation phenomena or to an improved colloidal stability in natural waters [22], thus significantly influencing their mobility and determining their association with different environmental compartments. The former case can result in faster sedimentation phenomena [23], with ENMs likely ending up in the sediment-associated fractions. However, adsorption onto organic or inorganic colloids or coating by polysaccharides or humic/fulvic substances, in some cases can improve steric stabilization of ENMs [24,25], causing them to travel longer distances in the water column leading to an enhanced transport. Such distinct behavior stems from the complex interplay between different water chemistries, such as multivalent cations, natural colloids, and DOM, and ENMs properties, governing their environmental partition, which rebounds on exposure and hazard levels and ultimately ecotoxicity [17,26,27]. Therefore, ENMs behavior pose questions regarding their environmental fate and impact after release in the environment, beyond the envisaged benefits in terms of contaminant removal or degradation, with consequent environmental costs [28]. Indeed, one key issue is the possibility to retrieve and/or remove ENMs, after they have exerted their action. This is to some extent possible with magnetic ENMs, such as magnetite nanoparticles, which can be recovered with the application of weak electromagnetic fields once the remediation process is over [29]. Nevertheless, major challenges are experienced with the vast majority of non-magnetic ENMs, as an effective removal from remediated environmental media is often limited or completely impractical. To this end, efforts to assess and model the fate of different ENMs in a wide range of environmental matrices, and to track relevant physico-chemical transformations, are much needed to anticipate potential endpoints [28,30,31,32,33]. Additionally, the complexity of environmental matrices requires novel and tailored detection and characterization technologies and strategies [34].

Potential ENMs bioaccumulation due to ingestion, dermal contact, and inhalation in wildlife is still unknown as well as their potential role to act as a *Trojan horse* by increasing the uptake of contaminants to be remediated in exposed organisms [35,36,37,38]. Far more important, the current technical limitations in ENMs detection in environmental matrices as well as a proper risk assessment procedure are still challenging and limiting its development worldwide [39].

It is hence wise to foresee possible scenarios of ENMs interactions with natural ecosystems and to screen for their potential ecotoxicity toward different levels of biological organization [17,40,41,42]. In this light, adaptations of existing ecotoxicity tests, together with ad hoc testing strategies for nanomaterials, have been recently developed and recommended [43,44,45,46,47]. A recent body of literature has been produced over the past few years concerning the hazard posed by ENMs and different nanoformulations actually employed for nanoremediation purposes, as reported in Table 1. Indeed, for most of the applied nanoscale materials in nanoremediation, several adverse effects in both terrestrial and aquatic organisms have been reported, thus certainly increasing governmental as well as public concerns related to their in situ application [48,49] (see Table 1). Among the tested ENMs, nZVI and iron oxides-based formulations received much attention, compared to other ENMs types, due to their consistent usage in ground- and surface water remediation [50]. Hjorth and co-workers [48] tested different commercially available engineered Fe nanomaterials, including nZVI, Fe oxides and hybrid products made of iron-carbon and iron-aluminosilicate nanocomposites, at concentrations close to environmentally realistic usage scenarios and in some cases below the concentration of 100 mg/L, which is, according to EU regulations, the baseline concentration for environmental hazard labeling of chemicals. Interestingly, among the tested ENMs, a ball-milled nZVI caused significant toxicity below 100 mg/L, affecting *E. coli* bacterial growth, *R. sativus* root elongation and increasing *L. variegatus* mortality, and the authors hypothesized ROS mediated damages as the main toxicity driver. Conversely, hybrid iron ENMs were not found toxic up to 100 mg/L. Keller et al. [51] proposed the release of Fe^2+^ and Fe^3+^ ions, together with oxidative stress, as the main cause of the remarkable toxicity caused by different nZVI ENMs toward fresh and seawater phytoplankton and the crustacean *D. magna*. Similarly, in a more recent study from Nguyen et al. [49], nZVI seems responsible for conspicuous ROS generation in the unicellular green alga *Chlamydomonas* sp., compared to other tested non-zerovalent Fe nanomaterials such as Fe-zeolites and Nano-Goethite, or hybrid materials such as Carbon-Iron, which generated much lower, yet significant, ROS levels. Other phytoplankton functional endpoints were affected by Fe ENMs exposure, such as the photosystem II quantum yield, Chlorophyll a content, cell growth rate and cell membrane damage, with the nZVI being more toxic compared to the other tested ENMs. The author linked such toxicity trends to the content of Fe(0) that caused high release of Fe(II) and Fe(III), which could be in turn be taken up by cells causing oxidative stress [52,53]. On the other hand, such mechanism was to some extent limited concerning the other tested material, due to surface passivation or absence of zerovalent iron in the formulation [49]. Oxidative stress and ROS generation has been recognized as the main cause of toxicity induced by titanium oxides ENMs as well [54]. TiO_2_ nanomaterials are popular photocatalysts employed in the remediation of polluted surface- or groundwaters and for wastewater treatments, by enhancing the photodegradation of organic contaminants and promoting water disinfection [55]. Miller et al. [56] demonstrated that, under realistic levels of ultraviolet radiation, the toxicity of TiO_2_ NPs is exacerbated toward three out of four tested marine microalgae species, compared to UV-blocked treatment, significantly inhibiting cell growth rates. This was due to an overall increase in ROS production in seawater contaminated with TiO_2_ NPs, which can deeply affect phytoplankton primary producers and compromise ecosystem functionality. Loss of membrane integrity and decrease in cell viability were identified by Mathur et al. [57] as the main toxic effects toward the bacterium *M. caseolyticus* exposed to low dose of TiO_2_ NPs, with such effects being more pronounced under ultraviolet (UV) A radiation. However, the authors demonstrated that TiO_2_ NPs can interact with bacterial biofilms, pointing out that ENMs trapping by exopolymeric substances could, to some extent, decrease their mobility and potentially play a role in modulating their toxicity. In a 2017 review, Callaghan and MacCormack [58] gathered abundant data regarding the lack of acute mortality of common TiO_2_ nanoformulations toward different fish species exposed under environmentally realistic conditions. However, the authors highlighted how, under chronic exposure, TiO_2_ ENMs promoted diverse physiological alterations, ranging from gill histopathology and brain dysfunctions to swimming impairment. Similarly, zinc oxide (ZnO) ENMs are excellent photocatalysts that hold promises in nanoremediation of polluted water bodies via degradation of organic pollutants, such as endocrine disrupting compounds [59,60]. Therefore, the main toxicity mechanism proposed for ZnO ENMs is oxidative stress promoted by ROS production [61]. However, differently from TiO_2_ NP, ZnO-based nanoformulation are soluble in water, and since nanoparticles have higher surface area to volume ratios than bulk counterparts they often display faster dissolution, making the release of zinc ions and zinc hydroxides [62] a primary concern for ecotoxicology [63]. Indeed, Miller et al. [64] showed that, without enhanced UV illumination, ZnO NPs significantly decreased the growth rate of four different marine phytoplankton species, possibly due to dissolution phenomena, while TiO_2_ NPs caused negligible effects under the same exposure conditions. Other studies [65] confirmed such trend reporting ZnO NPs toxicity to the freshwater microalga *P. subcapitata,* also showing very similar no-observed-effect-concentrations for both nano- and bulk ZnO*.* The absence of a nanospecific effect in promoting ZnO ENMs toxicity was also confirmed by Mortimer and co-workers [66] who gathered similar EC_50_ values for bulk and nanosized ZnO and Zn^2+^ ions toward the ciliated protozoa *T. thermophila*. 

Carbon nanotubes (CNT), either single- or multi-walled, have been successfully exploited in many technological fields, including wastewater treatment [67]. Therefore, this class of carbon-based ENMs are currently being released in the environment. Concerns have been raised about their environmental behavior and impacts on living organisms [68] and some ecotoxicological evidences have been produced regarding their effects toward different organisms. Hanna and co-workers [69] showed that CNT can be accumulated in the tissues of exposed marine mussels and can decrease phytoplankton clearance rate at low concentrations, while higher concentration can elicit toxic responses. Moreover, to date, there is no shortage of data regarding the toxic effects of CNT on both fish and crustacean species, as highlighted in a recent review from Callaghan and MacCormack [58]. DNA damage and ROS formation have been proposed as the primary drivers of CNT-induced toxicity in model organisms and the authors pointed out that interactions of CNT with organic and inorganic “classical” pollutants are an important factor in carbon nanotubes toxicity assessment. Indeed, Boncel et al. [70] evidenced the high adsorption capacity of CNT toward both heavy metals and hydrocarbons, pointing out that *Trojan horse* effects are likely to occur when they are discharged in previously contaminated water bodies.

Moreover, such effects have been described over different level of biological organizations, ranging from plants [71] and algae [49] to aquatic invertebrate and vertebrate species [48,72], identifying diverse toxicological endpoints. Such evidences highlight the necessity to move toward different nanoformulations and usage strategies when applying ENMs and ENMs-based products to natural waters.

**Figure 1:**
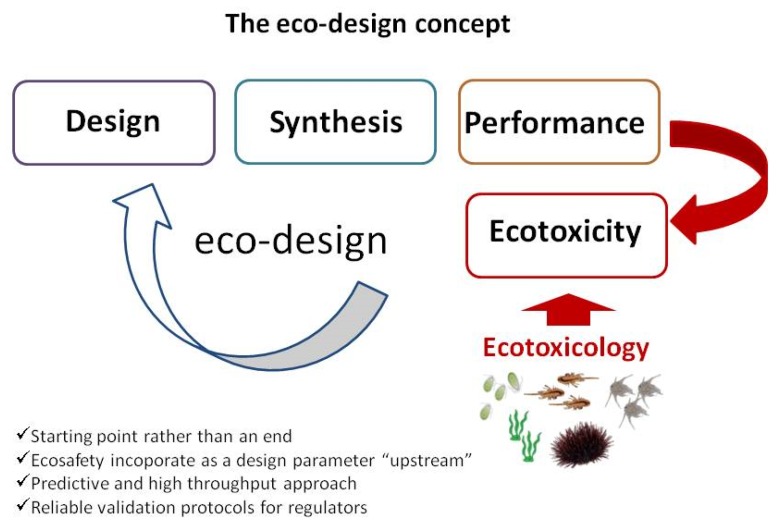
Schematic description of role of ecotoxicology in defining eco-design along ENM synthesis and development.

The “safety by design” concept is not new in nanotoxicology and it has been widely applied in other sectors (drug delivery and development) [73]. It is based on avoiding those undesirable properties of ENMs, which turn to be hazardous for environment and human health, in the process of ENMs design. Only those properties which will maintain ENMs efficacy and safety should be incorporated as a design parameter during product development. Environmental risk assessment of ENMs should provide suitable ecotoxicity data in terms of exposure and effects to non-target organisms which will help to recognize those ENMs properties as for instance behavior and transformations in environmental media which could affect interaction with living organisms and consequently toxicity. Such knowledge should be used to select only those properties of ENMs which will guarantee their ecofriendly and sustainable application also for environmental remediation [32]. Therefore, an eco-design of ENMs for environmental application obtained from an ecotoxicological testing strategy will allow the selection of the best ecofriendly and ecological sustainable ENMs and will significantly limit any potential side effects in term of no toxicological risk for natural ecosystems. A thorough ecosafe predictive assessment approach is proposed based on the following key aspects: gather information on ENMs behavior in environmental media to be remediated in terms of physico/chemical transformations occurring, which might affect their reactivity and fate and identify potential ENMs biological targets and provide a mechanism-based assessment of ecotoxicity from single model species up to ecosystem level (from microcosm to mesocosm and in situ studies). Upon observed ecotoxicity, ENMs should be modified up to become ecosafe, also by defining their behavior and transformation once released into the natural environment (Figure 1). Standardized methodologies able to assess ENMs effectiveness, environmental safety, and economic sustainability within the context of existing environmental regulations are thus urgently needed. All these aspects will certainly support patenting and pilot applications of new ENMs developed based on ecosafety by design approach.

## 3. Life-Cycle Assessment of ENMs

The progressive diffusion of ENMs in many fields, including nanoremediation, and the global consensus that their release into the environment will increase, has led not only to the urge of a sound evaluation of their toxicity effects on human health and on the environment, but also to the need for the evaluation of their environmental sustainability.

Life-cycle assessment (LCA) is a well-established tool, nowadays largely used to evaluate the potential environmental impacts of a product system (product or service) over its whole life cycle, from the extraction and acquisition of raw materials, to the core production process, use and end of life treatment, either recovery or final disposal [79,80].

The method consists in the compilation and evaluation of the inputs, outputs, and the potential environmental impacts of a product system throughout its entire life cycle, ”from cradle to grave”. LCA is considered a holistic method since it provides the assessment of the potential environmental impacts on several environmental categories, mainly on global and regional scale, such as global warming potential, ozone depletion potential, acidification potential, resource depletion etc. Moreover, LCA permits to define the environmental hotspots of a product system, to analyze alternative solutions that provide performance improvement and to make comparison of different scenarios, therefore proving to be a powerful tool for supporting eco-design and decision-making.

LCA is deemed to be the suitable tool to assess the environmental impacts of emerging technologies such as nanotechnologies and nano-enabled products, also in comparison to conventional technologies [81,82,83]. This application is largely debated in the scientific literature and at the same time is present also in policy documents [84,85].

Although LCA is strongly recommended as tool to assess the sustainability of ENMs throughout their life cycle, the scientific community currently agrees on the several information gaps, which hamper the proper application of LCA in the field [86,87]. These gaps regard mainly two broad issues, namely the difficulty of including the whole life cycle of ENMs and to fully assess their impacts on human toxicity and ecotoxicity. The first issue stems from the lack, in the life-cycle inventory, of specific features and properties of the new nanomaterials that differentiate them from the corresponding bulk material and of the quantification of their release into the environment across their life-cycle [84]. The second issue, instead, regards the application of the impact assessment methods, which currently do not allow consideration of the nanospecific impacts on human health and ecotoxicity. In fact, on the one hand the fate of ENMs in the environment is poorly modeled and quantitatively assessed and on the other hand, current impact assessment tools (e.g., USEtox^TM^) still lack to consider nanospecific properties and thus are not able to provide suitable characterization factors to include them in the assessment of impacts on health and environment.

Several review articles on the application of LCA to nanotechnologies and ENMs have been published that show the main gaps that prevent the majority of the LCA studies to be considered fully comprehensive [81,84,85,86,87,88]. The main conclusions of these reviews can be summarized as follows. Studies regarding LCA and ENMs are not very abundant, although increasing in the recent years, as to show the growing interest in the issue. However most of them mention the barriers and the challenges in the application of LCA on ENMs, but do not provide concrete solutions to overcome them. For instance, many studies focus only on the production stage, from cradle to gate, leaving out the use and end of life stages, mostly due to scarce availability of data regarding the potential release and fate of ENMs to and into the environment during these stages [81,84,88]. Consequently, the choice of the functional unit is often weight-based (e.g., 1 kg), instead of based on the functionality provided. This approach prevents the valorization of the augmented functionalities typical of ENMs that improve their performance in the use stage. This can be particularly significant in case of comparison with bulk materials, since the inclusion of the use stage would allow mitigation of the large impacts often found in the production stage of nanomaterials [68,81].

Therefore, to perform cradle-to-grave analyses, scholars agree on the urge of populating the inventory data of ENMs, regarding the release to environment compartments in the different life-cycle stages: in the production stage (release in the environment, workers exposure), in the use stage (planned release or unintentional release to the different environmental matrices), and in the end of life stage (e.g., in incineration or landfilling). As long as the assessment of such emissions release is not established by the scientific community, data regarding physico-chemical features of ENMs should be included in the inventory (e.g., particle number, the size distribution, surface charge, surface composition etc.). In particular, information regarding shape, dimension, properties, and surface chemistry, which are known to affect the interaction with the environment, are required to distinguish material flows of nanoproducts from bulk materials [68,88]. Since the definition of this information is very challenging, some authors suggest that, if scalability exists, it is then possible to apply traditional characterization approach, using the corresponding bulk emissions in the Life Cycle Inventory (LCI) and the same toxicity assessment approaches to determine the characterization factors [81].

The second critical issue regards the lack of suitable characterization factors (CFs) for the indoor and outdoor release of ENMs, with the major consequence that ecotoxicological impacts are not adequately represented by current available impact assessment methods. The main reason relies on the fact that nanospecific fate, transport, and toxicity effects in the environmental compartments (air, water, and soil) are not fully known [80,83,84]. In this context, the integration with ecotoxicological studies is very important both for developing CFs and inventory data. However, the definition of suitable CFs for these impacts is still a main unsolved challenge. In fact, most of the LCA studies on ENMs currently provide only results on common impact categories such as global warming potential, Water depletion, Acidification potential etc., which do not account for the nanospecificity of the materials [89,90].

Different approaches have been pursued to include these nanospecific aspects in the impact assessment phase of LCA. There are a few attempts in the literature, each very specific for the material considered, to develop suitable CFs of ENMs for the toxicity impact categories (human and ecological toxicity). For instance, Walser et al. [91], based on literature data, modeled the environmental impacts of the release of silver nanoparticles with biocidal function from T-shirts. The CFs for the aquatic environment were calculated with the USES-LCA model, since only the bioavailable silver fraction from the wastewater treatment plant was considered relevant. Eckelman et al. [92] applied the USEtox^TM^ model to calculate the CFs for the freshwater ecotoxicity for carbon nanotubes. For a conservative scenario and a realistic scenario, they obtained a CF of 29,000 PAF m^3^ day kg^−1^ and of 3700 PAF m^3^ day kg^−1^, respectively. Salieri et al. [93] provided freshwater ecotoxicity CFs for titanium dioxide nanoparticles (nano-TiO_2_), by using USEtox^TM^ characterization model. The authors developed a specific multimedia fate model that includes specific nanospecific fate processes (i.e., sedimentation, aggregation with suspended particle matter, etc.) and applied a HC_50_ value calculated from EC_50_ values taken from the literature, to obtain a CF for the toxic impact of freshwater ecotoxicity of 0.28 PAF m^3^ day kg^−1^.

Another largely explored approach to include the impacts of ENMs on human health and the environment in sustainability assessment is the integration of LCA with other methodologies, such as Risk Assessment (RA). Although the two methods have different aim, they can be considered complementary in the evaluation of environmental impacts. Moreover, this integration can also guarantee the consideration of local critical specific parameters that would not be included in LCA evaluation [81].

Grieger et al. [82] identified two main approaches: life cycle-based RA (namely, traditional RA based on a life-cycle perspective) and combined use of RA and LCA (conventional LCA integrated by RA in specific life-cycle steps).

In the first approach, the challenge is to integrate the exposure assessment over the different life-cycle stages with the identification of the qualitative and quantitative characteristics of the release and their interaction with the environmental matrix. A few studies report this approach and they mostly agree that the exposure situations depend on the ENM nature and on the life-cycle stage and handling method [94,95].

In the combined use of LCA and RA, the approach is to apply the two methods separately. For instance, Barberio et al. [96] proposed a framework to combine the use of LCA and a qualitative RA and applied this to a case study on the production of nanofluid alumina. The combined use of both tools provides a more comprehensive evaluation of the impacts and better support a Safe-by-Design approach and the decision-making process.

The state of the art described so far leads us to the conclusion that LCA is certainly a valuable and powerful tool for the sustainability assessment of emerging technologies, even though several methodological issues need to be solved to provide a comprehensive assessment in the field of nanotechnologies. Indeed, its main limits will be overcome with the progress of the ecotoxicological characterization, as highlighted in the previous paragraph. Nevertheless, at the current stage, LCA can be fruitfully applied to the emerging technologies as an eco-design tool to identify the most impacting steps of the production stage and suggest alternatives to improve the environmental impacts not only at the lab scale but also in their transition from the laboratory scale to the semi industrial or industrial scale. Indeed, the application of life-cycle thinking (LCT) approach to the development of emerging nanotechnologies is strongly recommended at the early stage since it is evident that the technological maturity and scale of production affect the environmental impacts of the production processes [97]. Gavankar et al. investigated a case study on carbon nanotubes (CNTs) manufacturing showing that regardless of synthesis technique, CNT manufacturing becomes less energy intensive upon increasing technological readiness, thus reducing its environmental impacts. This confirms the relevance of the scale of production in life-cycle inventory practices.

In this sense LCA can support the development of Green Nanotechnology, namely the implementation of cleaner and greener production methods based on synthetic strategies without using raw materials, containing scarce natural resources or hazardous substances, and applying production methods that require low energy and resources (e.g., water) consumption [98].

However, the application of LCA should always include the whole life cycle of the nano-product to provide a complete picture of the sustainability of the product, without overestimating or underestimating the environmental impacts generated in the different life cycle stages. Dhingra et al. report a case study on an automotive exterior body panels, which shows that the perceived environmental benefits of nano-based products in the use stage may be overestimated, without examining the impacts in the other life-cycle stages, particularly Materials Processing and Manufacturing [99]. As also evidenced by Carpenter et al. [100], we consider the evaluation of the life-cycle environmental impacts of ENMs materials particularly important in water and soil remediation applications, where large quantities of nanostructured materials are likely to be required

## 4. Cellulose- and Starch-Based Nanostructured Materials

Even if, at the beginning of this millennium, application of nanotechnology to environmental remediation was expected to grow rapidly [11], we should recognize that the process of its implementation at full, or at least pilot scale is limited to a few examples, mostly related to the use of zero-valent iron nanoparticles (nZVI) [6,101]. Indeed, ongoing innovation pursued by scientific community continuously exploits the potential advantages that could be achieved by using ENMs in water and soil treatment [5,101]. However, as recently claimed by Bartke et al. [102], “any new technology has to prove that is complementing or improving existing technologies, at an appropriate economic cost and acceptable risks” [102]. Nanotechnology applied to environmental remediation cannot represent an exception. On the contrary, more than other approaches, it suffers from the uncertainty about the fate of ENMs, with the consequent distrust of society. For this reason, besides the ongoing progress on nZVI [103,104,105,106] and iron oxide nanoparticles [107], new materials are emerging in the field of water nano-treatment, aiming to combine the advantages of nanotechnology with the use of renewable, sustainable, and ecofriendly sources, possibly derived from waste production, in accordance with the virtuous supply chain of circular economy. Moreover, the switching from nanosized systems to nanostructured micro-dimensioned scaffolds could allow overcoming of the potential risks related to the use of ENMs, while taking advantage of the enhanced performances obtained by operating in the nanoscale dimension [46]. In our opinion, following this approach would represent a first, although still not decisive step toward the development of ecosafe solutions for water decontamination. In this context, cellulose and starch derivatives have attracted our attention as valuable starting materials, meeting most of the requirements above mentioned.

Starch is an important and sustainable natural source, from which it is possible to obtain a variety of products of industrial interest, following either chemical or biochemical approaches [108]. Among the wide range of possible transformations, enzymatic degradation of starch by bacteria leads to the formation of cyclodextrins (CDs), which are cyclic oligosaccharides containing 6 (α-CD), 7 (β-CD), or 8 (γ-CD) glucose units. These macrocyclic entities, shaped like cones, display a geometrically well-defined hydrophobic central cavity and a hydrophilic external surface. This intrinsic characteristic gives the CDs molecular recognition and complexation properties [109]. For this reason, CDs have found ample application in the field of drug delivery and in cosmetics [110,111]. The host-guest interactions with apolar and polar molecules can be modulated by functionalization on the secondary hydroxyl groups extending from the wider edge, or by the formation of CD-metal complexes and CD-inorganic nanoparticle systems [112]. Moreover, selective grafting on the same hydroxyl moieties can be used to introduce additional properties for advanced applications. As examples, we have recently proposed 2,2,6,6-tetramethylpiperidinyloxy (TEMPO)-labeled β-CDs as potential supramolecular vectors for magnetic resonance imaging [113,114], and *N*-hydroxyphthalimide (NHPI)-labeled β-CDs as suitable supramolecular pro-oxidant organocatalysts [115] for regulation of oxidative stress, taking advantage of their high biocompatibility and negligible toxicity. More recently CDs have been also proposed as suitable systems for water remediation, even if, being partially soluble in water, they need to be immobilized on appropriate supports or to be cross-linked, to exploit their adsorption properties [116]. For example, water insoluble β-CD-epichlorohydrin (β-CD-EPI) polymers have been widely investigated in sorption processes for the removal of contaminants from water solutions [117]. However, cross-linkers such as EPI or glutaraldehyde (GLA) have been reported to be highly toxic for humans and animals [118,119]. A few years ago, Trotta et al. [120,121] developed and patented a new class of highly cross-linked CD polymers, referred to as CD nanosponges (CDNS). CDNS can be obtained following one-step synthetic protocols by reacting CDs (and in particular β-CDs) with polyfunctional cross-linkers, such as pyromellitic dianhydride (PMA), carbonyl diimidazole (CDI), and the dianhydride derived from ethylenediamine-tetracetic acid (EDTA) [122].

In recent years, with the synergistic cooperation of different research groups, we have contributed to define the nanoporous structure of PMA- and EDTA-CDNS by the combined use of different and complementary techniques, such as Fourier transform infrared absorption in attenuated total reflectance geometry (FTIR-ATR) and Raman spectroscopies [123,124,125,126,127,128,129], high resolution magic angle spinning (HR-MAS) [130,131] and solid-state NMR spectroscopy [132], and Small Angle Neutron Scattering (SANS) experiments [133]. With a network of both CD hydrophobic cavities and more hydrophilic channels, these nanosponges show versatile adsorption properties, extended to both heavy metal ions and organic pollutants. Indeed, in 2015 Zhao et al. reported this bifunctional adsorbent behavior for EDTA-CDNS, indicating an adsorption capacity of 1.241 and 1.106 mmol g^−1^ for Cu(II) and Cd(II), respectively, and a heterogeneous adsorption capacity of 0.262, 0.169, and 0.280 mmol g^−1^ for Methylene Blue, Safranin O, and Crystal Violet, respectively [134]. These excellent adsorption performances, combined with their safety for human beings and environment, render CDNS valuable examples of nanostructured systems, and led us to further implement their formulation in NanoBonD project, to produce new, more efficient materials for water treatment. However, these materials will not be further discussed in the following sections. Another renewable source of relevant interest is cellulose. It is a biodegradable and biocompatible carbohydrate polymer consisting of β-d-glucopyranose units. The favorable combination of both physic-mechanical features and chemical ones, the latter due to the high concentration of functional hydroxyl groups on the macromolecule backbone, has made this natural source highly attractive for the design and development of several organic devices. In particular, it is possible to cleave the hierarchical structure of native cellulose via mechanical or chemical approaches, promoting nanofibrillation and consequent production of nanocellulose (NC), in the form of cellulose nanocrystals (CNCs) or cellulose nanofibers (CNFs).

NC has been widely investigated in the last decade [135,136], and its use has been suggested for different applications, including food science, packaging, catalysis, development of energy storage devices. No less important, sustainable cellulose nanomaterials are becoming increasingly attractive also for their potential use in environmental remediation and water/wastewater processes, such as sorption, membrane filtration, catalytic degradation, and disinfection. Both CNCs and CNFs have been widely investigated for this purpose, and very recent reviews furnish a detailed state of the art based on a wide overview of the literature [100,137,138,139,140,141,142].

**Figure 2:**
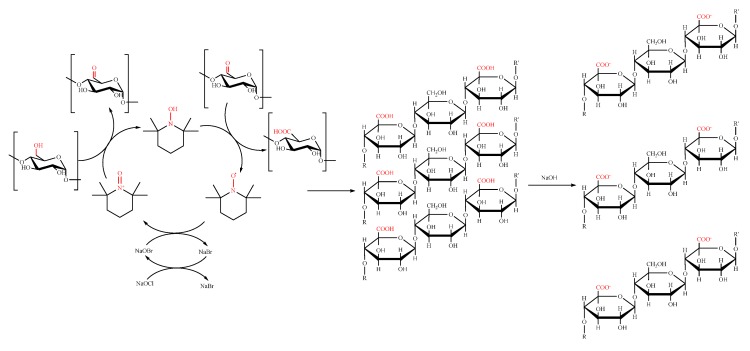
Mechanism for the TEMPO-mediated oxidation of cellulose fibers.

CNCs are usually obtained by acidic hydrolysis of cellulose fibers. Their absorption properties are mainly ascribed to the interaction of the negative charges, introduced during the hydrolysis process, with a wide range of transition metals and of organic dyes positively charged. Moreover, they can be further functionalized, for example by introducing carboxylic groups, with the aim of increasing their adsorption properties, or amino-moieties, to extend their interaction capability to negatively charged systems. More recently, they have been also proposed as stabilizers and delivery vehicles of nZVI for groundwater remediation, providing colloidal suspension with high mobility [142]. However, the synthesis of CNCs requires aggressive conditions to disintegrate the amorphous regions of pristine cellulose fibers. This implies a loss of starting material, with consequent low atom-economy efficiency of the process, which could affect implementation at industrial scale. For this reason, CNFs can be considered even more attractive. They can be obtained by simple mechanical disintegration of plant cellulose fibers in water, even if this technique leads to the damage of nanofiber structures. An alternative route to produce CNFs consists into the oxidation of cellulose pulp. Among the several protocols reported in the literature for this purpose, the most investigated one is the TEMPO-mediated oxidation by NaClO/NaBr system [143,144]. Following this approach, it is possible to partially convert the alcoholic groups in the C6 position of the glucopyranosic units to the corresponding carboxylic acids. Moving at basic pH, the deprotonation of carboxylic groups occurs, introducing negative charges on the backbone of the single nanofibers, and favoring the physical nano-defibrillation of the cellulose hierarchical structure by simple electrostatic repulsion between CNFs (Figure 2). Consequently, if the oxidation occurs under controlled and mild conditions, it is possible to obtain CNFs with diameters of 5–100 nm and lengths up to 5 μm, characterized by a high surface area and good mechanical strength.

## 5. The NanoBonD Action

In accordance with the requirements above mentioned for a sustainable nanotechnology, both from the environmental and the economic point of view, we considered the TEMPO-mediated oxidation of cellulose as the most promising technique for the first step in the design of a scalable and ecosafe material for water treatment. Moreover, the introduction of carboxylic units on CNFs backbone also provides linking hooks for further grafting and/or reticulation, without requiring intermediate chemical reactions. The cross-linking of CNFs becomes a crucial step for the design of nanostructured units, overcoming the derived limits by the use of nanosized particles in the environment.

**Figure 3:**
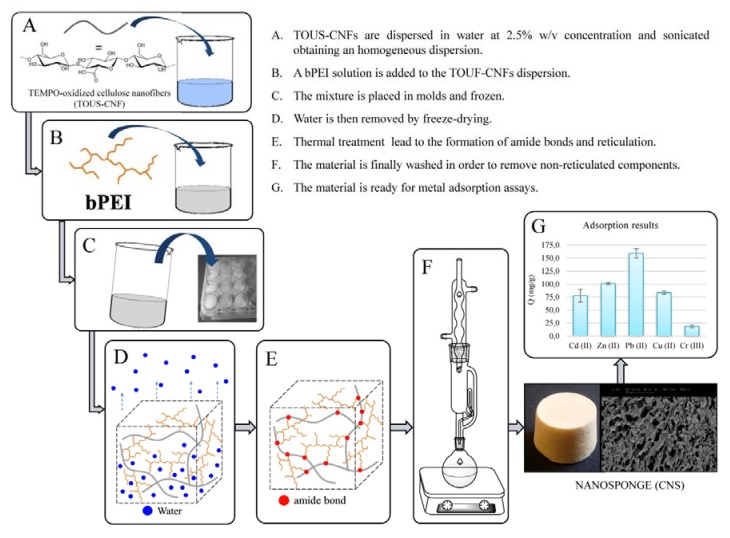
Synthetic process of TOUS-CNFs/bPEI adsorbent nanosponges.

Following this route, in 2015 we reported a thermal protocol for the one-pot cross-linking of TEMPO-oxidized and ultra-sonicated (TOUS) CNFs, putting the bases for a new composite material further developed within the NanoBonD project (Figure 3) [145]. The synthesis was inspired by the freeze-drying process previously reported for the formulation of CNF-based cellular solids [146], which we also used as templates for the design of ceramic [147] and eumelanin-coated organic xerogels [148]. Briefly, a hydrogel of TOUS-CNFs is mixed with water solutions of branched polyethyleneimine (bPEI). The resulting gels are put in molds, frozen, and dried by sublimation, affording the corresponding aerogels, whose porosity derives from the ice-templating action of water. The new materials disaggregate in water. However, a simple thermal treatment at about 100 °C promotes the formation of amide bonds between the carboxylic groups of TOUS-CNFs and the primary amines of the polycationic polymer. The result is a nanostructured aerogel (cellulose nanosponge (CNS)) exploiting superb efficiency in the removal, by adsorption, of a wide range of contaminants from Milli-Q water. Figure 3G shows the adsorption performance of CNS (1.2 G L^−1^) from a 150 ppm Milli-Q water solution of a wide range of heavy metals. The capture efficiency results to be good for Cu(II) (84 mg/g) and Cd(II) (77 mg/g), and even better for Zn(II) (101 mg/g) and Pb(II) (160 mg/g), while adsorption of Cr(III) is less effective (18 mg/g) (unpublished results). Moreover, CNS have a bifunctional adsorbent behavior, as we demonstrated their capture efficiency also toward representative phenols, used as precursors in the synthesis of several drugs and fungicides, and emerging contaminants such as amoxicillin [145].

Since our first report, several approaches have been reported for promoting the cross-linking between NC and bPEI, with the aim of synthesizing efficient composite adsorbents for heavy metal ions, all of them requiring chemical co-cross-linkers and/or activators. In 2016, Ge et al. [149] suggested the use of a “one-step” method to synthesize composite cellulose/PEI hydrogels in LiOH/urea water solution, by using EPI as cross-linker. The obtained material resulted particularly efficient for the adsorption of Cu(II) and other metal ions [149]. In the same year, Zhang and co-workers reported a TOUS-CNFs/bPEI reticulation protocol, promoted by GLA. This process required the pre-hydrolysis of cellulose fibers with HCl solution at 80 °C before the oxidation step, and the use of methanol solvent in the cross-linking phase [150]. However, as previously discussed, EPI and GLA do not represent the best choice in terms ecotoxicity and human safety. More recently, a material very similar to that developed by our group was described by Zhao et al. [151] to be an efficient system for rare earth elements recovery, such as La(III), Eu (III), and Er (III). However, in this case the formation of the amide bonds was promoted by the activation of carboxylic acids and amines using *N*-(3-dimethylamino)propyl)-*N′*-ethylcarbodiimide hydrochloride (EDC) and *N*-hydroxysuccinimide (NHS), with TOUS-CNCs: EDC and TOUS-CNCs: NHS 1:1 ratios (*w*/*w*). Moreover, according to this procedure, oxidation was conducted onto pre-formed and commercially available CNCs, while the purification step required cycles of dialysis. Compared to these procedures, in our protocol cellulose pulp oxidation represents the unique chemical step of the entire process, rendering this approach commercially appealing and easily scalable. Moreover, we have demonstrated that this synthetic approach could be conducted using pre-functionalized bPEI, without affecting the sponge-like morphology and the chemical stability of the composite but introducing additional properties to the adsorbent. As proof of concept we reported the cross-linking of TOUS-CNFs with bPEI grafted with *p*NO_2_-phenyl urea, achieving an aerogel which was successfully used for the heterogeneous sensing of fluoride anions in DMSO solution [152]. More recently, we have also suggested a smart strategy to increase the content of carboxylic moieties in the hydrogel formulation, without excessively stress the oxidation of pristine cellulose. In fact, it has been verified how the oxidation conversion is limited, even when NaClO is used in large excess. Nevertheless, a higher number of carboxylic groups would allow increase of cross-linking, maximizing the yield of the synthetic process. For this reason, we suggested the introduction of citric acid as co-crosslinker in the formulation to be freeze-dried. This simple, till sustainable modification allowed increase of both the mechanical and the chemical stability of the new adsorbents, without affecting its characteristic of eco-sustainability [153].

Although the choice of a renewable starting material, the selection of a cheap and scalable synthetic process, and the high efficiency in the removal of contaminants and pollutants from water are crucial criteria toward the full-scale implementation, all these aspects are not sufficient by themselves the for validation of this new nanotechnology which, due to its final application, also requires high standards of ecosafety. To reach this goal, within the NanoBonD project both the formulation and the synthetic process have been further modified and optimized, following an eco-design approach and the guideline of a detailed life-cycle assessment analysis.

Moreover, in a long-term perspective, since most water and soil remediation applications are likely to require large quantities of ENMs, the cost, feasibility, and life-cycle considerations of manufacturing these materials on a large scale must be considered, as also evidenced by Carpenter et al. [100].

**Figure 4:**
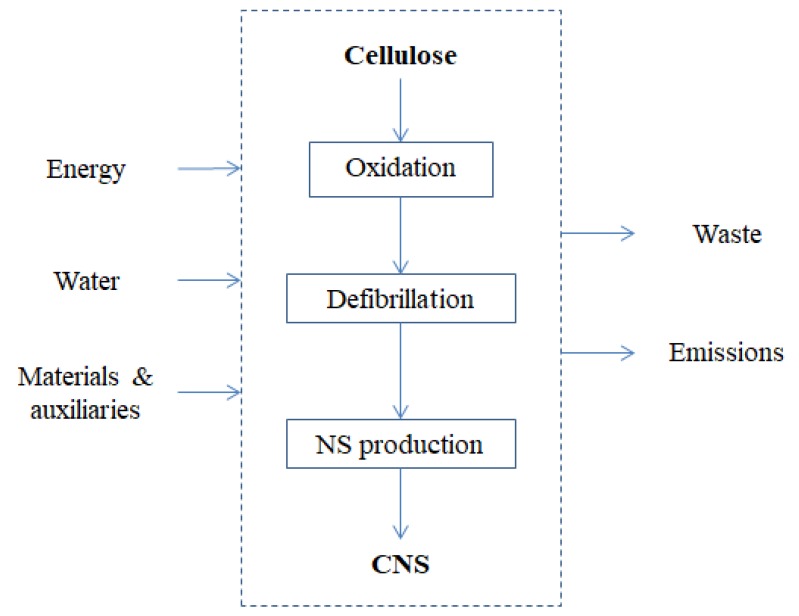
System boundaries and input and output flows considered in the LCA analysis of CNS.

LCA was applied since the early stage of the development of the synthetic process of the CNFs nanosponges, analyzing the inputs and outputs of each process step, regarding energy and material flows, emissions, and waste (Figure 4).

The specific purpose was to identify the environmental hotspots and provide basis for environmental improvement actions. Starting from the lab scale process, the main hotspots were found in the energy and water consuming steps, the raw materials and the solvents used in the washing steps, which impact not only in the resource consumption but also in the waste disposal stage. Therefore, actions to decrease the impacts of these categories were undertaken and an optimized synthetic strategy was proposed regarding mostly (i) the change of formulation ratio among cellulose, bPEI and citric acid, favoring higher yields in CNS; (ii) the change of washing solvents from organic (methanol) to aqueous; and (iii) the conduction of the purification step under milder conditions, lowering energy consumption. The optimized process was again evaluated with LCA and the analysis showed interesting improvements on most of the environmental impact categories assessed (e.g., climate change, acidification, etc.). However, the results confirmed that major improvements will be achieved with the optimization of the energy consuming steps, likely to be achieved in a scale-up configuration.

This approach made the overall synthetic protocol suitable for further scale-up. Indeed, the TEMPO/KBr/NaClO system is widely used for different industrial processes and, within the NanoBonD project, it has been possible to apply this oxidative approach in the conversion of different sources of cellulose at pilot scale, managing production of final CNS material in the order of kilograms for in-field demonstrative treatments on real matrices. However, implementation of the synthetic protocol is still under way to reduce the energy costs mainly associated to the lyophilization step.

A critical comparison of the proposed approach with the most consolidated technologies for wastewater treatment allows the outlining of advantages and limits in the use of CNS for water remediation, over the choice of a renewable and recyclable starting material. Among these we mention chemical precipitation (namely hydroxide, sulfide or chelating precipitation), the use of ion exchange resins or activated carbon, membrane filtration and electrochemical treatments [154,155,156]. As a general concept, it should be remarked that the selection of an optimal treatment technique would depend on the initial metal concentration. Processes such as chemical precipitation and electrochemical treatments are quite effective in the case of wastewater streams with high concentration of heavy metals but tend to fail at low concentrations of contaminants. On the other hand, adsorption, ion exchange and membrane filtration are effective at much lower concentrations but tend to be particularly expensive for treating large volumes [155].

In this context, the heavy metal removal efficiency of CNS is comparable to that obtained with different approaches for some analytes, while for other ones it must be still considered insufficient. However, the amount of adsorbent used per unit volume of water (1.2 g L^−1^) is at least an order of magnitude lower than that usually required by using standard adsorbents (i.e., ion exchange resins, zeolites and activated carbons) or membranes, and an increment in the adsorbent concentration might lead to further improvements in decontamination efficiency.

Moreover, most of standard technologies (e.g., precipitation and, in some cases, ultrafiltration and ion exchange resins) require a careful pH control and/or a specific pH to work at their optimum. On the contrary, CNS have been purposely designed to exploit their adsorption efficiency at pH = 7.6, also acting as buffer in different water matrices, so that pH adjustment is not required in field application.

Similar to activated carbons and ion exchange resins, CNS can be regenerated several times by counter-current washing with acidic solutions, maintaining good to excellent adsorption performances. However, being constituted by a bio-organic matrix, in this case the adsorbent material can eventually be disposed by combustion, while other technologies generate waste. 

Finally, further strengths of the use of CNS as adsorbent units for wastewater treatment are (i) their bifunctional action toward both organic and inorganic contaminants, while with standard approaches the coupling of different technologies is usually required; and (ii) their versatility for application in different water matrices, including marine water.

## 6. Conclusions and Outlook

Nanoremediation can provide enormous benefits and with appropriate strategies and (nano)solutions allowing reduction of uncertainties and environmental and human risks, they will satisfy regulatory requirements, boost circular economy, and support a fully effective deployment of the sector. Main recommendations are that ecosafety obtained by an eco-design approach should be recognized as a priority feature. Ecotoxicity testing should be more ecologically grounded and include more realistic environmental scenarios. Research and innovation should focus on greener, sustainable, and smart (nano)solutions with the aim of providing a more ecofriendly nanoremediation.

Regarding the environmental sustainability assessment of ENMs, it is acknowledged that LCA is a valuable tool to implement the LCT strategy. However, the full implementation is far from being feasible due to many unsolved methodological issues, which will very likely progress in parallel with the ecotoxicological characterization.

Nevertheless, at present, LCA can provide sound support for the eco-design of emerging ENMs, which is very important already at the early stage of their development, at the lab scale, and can guide usefully the transition to the scale-up phase. The experience carried out in the NanoBonD project confirms the validity of this approach. 

## Figures and Tables

**Table 1:** Documented ecotoxicity of selected ENMs for environmental remediation.

Nanoparticles Type	Remediation Mechanism	Remediated Contaminants	Potential Toxicity	Test Organisms	Reference
nZVI	Adsorption; oxidation; reduction	metals; chlorinated pollutants	Algal growth inhibition; ROS generation; oxidative stress; disruption of membrane integrity; genotoxicity; morphological alterations of roots; oxygen consumption	bacteria; freshwater microalga; freshwater crustaceans; earthworm; plant	[48,49,74,75]
Iron-based ENMs	Adsorption; oxidation; reduction	metals; microbiological contaminants	Algal growth inhibition; ROS generation; oxidative stress; disruption of membrane integrity; genotoxicity; mutagenicity; reproduction impairment	bacteria; freshwater microalga; freshwater crustaceans; earthworm; plant; fish	[48,49,72]
TiO_2_	Photodegradation	organic contaminants	ROS generation; oxidative stress; membrane damage; cell viability reduction; reproduction impairment; tissues alterations and gill histopathology; neurotoxicity	bacteria; crustacean; plant; fish	[57,58,60,76]
ZnO	Photocatalysis; photodegradation; adsorption	organic contaminants; heavy metals	Algal growth inhibition; ROS generation; gill damage; embryotoxicity; metal stress via dissolution and ion release; membrane damages	bacteria; freshwater microalga; crustacean; fish	[58,60,61,77]
CNT-based ENMs	Catalytic facilitation; adsorption	organic contaminants; heavy metals	toxicity enhancement of contaminants; carrying of pollutants; ROS generation; growth rate inhibition; membrane damage	bacteria; microalga; crustaceans; mollusks	[58,69,70,78]
